# Distribution of breast lesions diagnosed by cytology examination in symptomatic patients at Eritrean National Health Laboratory, Asmara, Eritrea: a retrospective study

**DOI:** 10.1186/s12905-020-01116-0

**Published:** 2020-11-10

**Authors:** Kidane Siele Embaye, Saud Mohammed Raja, Medhanie Haile Gebreyesus, Matiwos Araya Ghebrehiwet

**Affiliations:** 1 Department of Basic Medicine, Orotta School of Medicine and Dentistry, Asmara, Eritrea; 2 Department of Internal Medicine, Orotta School of Medicine and Dentistry, Asmara, Eritrea; 3 Department of Surgery, Orotta School of Medicine and Dentistry, Asmara, Eritrea; 4 Department of Allied Health Sciences, Asmara College of Health Sciences, Asmara, Eritrea

**Keywords:** Breast cancer, Distribution, Breast lesions, FNAC, Eritrea

## Abstract

**Background:**

Fine needle aspiration cytology is a simple, relatively accurate, non-invasive, and cost-effective method of diagnosing most breast pathologies. To date, there is no sufficient data depicting the distribution of breast lesions detected by fine needle aspiration cytology in our healthcare setting. The aim of this study was to elucidate the general distribution of breast lesions diagnosed by cytology test at Eritrean National Health Laboratory.

**Methods:**

This retrospective study was carried out on 905 symptomatic patients between the years 2013 and 2017 at Eritrean National Health Laboratory. Diagnosis was made by fine needle aspiration cytology in patients with palpable breast lump and in some patients direct smear was prepared from a nipple discharge. Statistical analysis was carried out using Statistical Package for the Social Sciences version 23.

**Results:**

A total of 905 patients were included in the study, of whom 871 (96.24%) were females. The age range of patients was from 13 to 93 years with mean and standard deviation of 33 ± 14.9 years. Breast lump, occurring in 892 (98.56%), was the most frequent presenting symptom. Fibroadenoma and fibrocystic breast lesions were the most prevalent lesions accounting for approximately 40% and 15%, respectively. Malignant breast lesions were seen predominantly in females above the age of 40 years with the highest frequency observed in the age range between 51 and 60 years. Pearson Chi-squared test showed significant association between patients’ age above 40 years and the risk of having a malignant breast lesion (*p* < 0.001). The highest number of benign and malignant breast lesions was documented in 2014 with little fluctuation elsewhere in the study period.

**Conclusion:**

Fine needle aspiration cytology is a procedure of choice for preoperative diagnosis in breast lesions mainly in a resource-limited settings. Our study identified the occurrence of malignant breast lesions in young women, which is of a paramount public health concern. Of note, significant proportion of patients were late to seek medical attention. Therefore, enhancement of community awareness regarding breast disease and implementation of screening programs are necessary to ameliorate the morbidity and mortality associated with the disease.

## Background

Global burden of breast cancer has posed a major public health concern with a steadily increasing occurrence worldwide [1, 2]. Malignant breast disorders are grave diseases and remain to be the leading cause of mortality and morbidity among women population [3, 4]. There is a high prevalence of neoplastic and non-neoplastic breast lesions in females of Sub-Saharan Africa region [5]. In Eritrea, a ten-year retrospective study done by Faisal M. et al. at Eritrean National Health Laboratory (ENHL) documented that the most common tumor diagnosed in female patients was breast cancer with age standardized incidence rate of 2.7 cases in every 100,000 living females [6].

The physical, psychological and financial costs of investigating benign breast disease with an intent to exclude malignancy are significant [7]. Common clinical symptoms of human mammary gland lesions include palpable breast lump, breast pain, and nipple discharge [5]. During evaluation of a breast lump, a physician is challenged as to whether the mass could be pathologic or normal variant of a breast tissue. On the other hand, the patient has a high degree of anxiety associated with underlying fear of breast malignancy even though the vast majority of breast lesions eventually end up to be benign [7, 8].

Triple assessments of breast lesions which consists of clinical, radiological and pathological evaluations are fundamental techniques to diagnose mammary gland disorders [9]. Hence, one of the current clinical approaches to palpable breast masses is to get cytopathologic evaluation before going to a definitive surgery. Apart from a patient’s own late presentation, delays in the diagnosis of breast cancer generally arise from a low index of suspicion by an assessing clinician. Excisional biopsy represents a traditional means of breast mass diagnosis which provides a relatively precise diagnostic impression, albeit unnecessarily invasive considering the likelihood of a benign pathological condition in many cases. On the contrary, fine needle aspiration cytology (FNAC) of the breast is deemed to be a minimally invasive, quick, cost-effective and easy to perform procedure often avoiding an open biopsy [3, 10–12].

Nearly 25 years ago, the National Consensus Institute meeting on cytology of breast lesions was held in the United States which intended to create a framework for technical as well as probabilistic reporting of breast FNAC [13, 14]. Ever since, there have been substantial modifications in the work up and management principles of breast lesions in cytology throughout the world. Recently in 2016, however, an International Academy of Cytology (IAC) Yokohama system for classification of breast lesions was established that provides a comprehensive approach of FNAC practice and reporting for breast lesions across the globe [15]. Experts from various specialties such as cytopathology, radiology, surgery and medical oncology were engaged in this international congress. According to the IAC classification system, there are five categories of cytological diagnoses for breast lesions: (1) Insufficient material, (2) Benign lesions, (3) Atypical lesions, (4) Suspicious for malignancy, and (5) Malignant lesions. Adopting a standardized reporting system maintains an improved FNAC practices and provides an excellent ground for quality assurance measures across various institutions. It also enhances quality, clarity and consistency of reports between centers both at national and international levels, thus leading to improved breast health care and quality of research [16].

Based on the increasing demand for cytology examination requests, and the day-to-day clinical experience of physicians in the country, both the prevalence and incidence of breast cancer are presumably rising in Eritrean women. Nevertheless, there is paucity of published data on the nature of breast cancer epidemiology, risk factors as well as overall disease distribution patterns. With these knowledge gaps in mind, this study was conducted in the pathology department of ENHL to evaluate the spectrum of breast lesions assessed by FNAC examination over a five-year period.

## Methods

### Study period and study participants

A total of 905 symptomatic patients were enrolled into the study after being referred by a surgeon or a treating physician for cytological evaluation of breast lesions over a period of 5 years from January 2013 to December 2017.

### Study design and study setting

A retrospective study was conducted to describe the general distribution of breast lesions diagnosed by fine needle aspiration cytology examination on symptomatic patients at ENHL. In Eritrea, FNAC is a commonly utilized diagnostic modality for patients with manually detectable breast masses. The ENHL is the highest and the nation’s only referral laboratory institution located in the capital city, Asmara. It officially started its function in the same year of the country’s independence as of early 1990s. Since then, the ENHL has been constantly working to upgrade the health laboratory services in the country. Being the central national reference laboratory, all kinds of cytohistopathology specimens from all regions of the country are routinely processed in this laboratory. The department of pathology at ENHL is a unit responsible for the evaluation of patients’ samples for both neoplastic and non-neoplastic disease entities nationwide.

### Inclusion and exclusion criteria

All patients with a breast complaint sent to ENHL for cytological test and their specimen got examined during the study period were included in the study regardless of their age and gender. Patients who presented only with a nipple discharge and diagnosed with direct slide smear preparation were also included. To avoid the possibility of metastasis from a previous lesion, patients with an already established diagnosis of breast cancer in the contralateral side were not included irrespective of their treatment status.

### Laboratory methods

#### Fine needle aspiration cytology

After proper counseling regarding the procedure and taking a written informed consent, all patients who came to ENHL with a surgeon’s or physician’s cytology request paper underwent fine needle aspiration sampling by an experienced cytohistopathologist. Briefly speaking, the skin overlying the breast mass was cleaned with antiseptic solution and the lump of interest was held firmly with the non-aspirating hand and the needle was inserted directly into it. The plunger of the syringe pistol was pulled back, thus exerting suction. This was maintained and the needle was made to move through the lump three or four times in different directions. With the needle still in the area of swelling, suction was slowly released. The needle was then removed from the breast lump and the syringe from the needle. The syringe was then filled with a little of air, reconnected to the needle, and the contents of the needle were blown on to one or more clean dry slides, which were rapidly air-dried.

After proper air-drying, the slides were stained using Romanowsky staining technique (May Grünwald Giemsa Stain). Subsequently, microscopic evaluation was done at two levels by a senior cytopathologist. At first, a low-power evaluation at 100× or 200× total magnification was carried out to assess cellularity, cell arrangements, and background elements. Later on, a high-power evaluation at 400×, 600× total magnification or oil emersion was performed in order to visualize; types of isolated cells, nuclear characteristics and intracytoplasmic details. Finally, a specific diagnostic impression or in some cases a possible differential diagnosis, together with its IAC category at the heading was provided in the report of FNAC.

#### Reporting standards of FNAC results

At the ENHL, a routine FNAC report for breast lesions includes a brief description of macroscopic and microscopic details of the lesion with its final specific diagnosis (for instance, ‘fibroadenoma’). If the cytopathologist is uncertain about the specific diagnosis, the most likely differential diagnosis is provided. In the present study, breast lesions were considered according to their specific diagnoses, and further classified into five categories as inadequate material, benign, atypical, suspicious and malignant based on the IAC Yokohama classification system [15].

Recent studies [17, 18] indicated that the Risk of Malignancy (ROM) for the first three categories (insufficient material, benign and atypical) is low (ROM = 2.6–15%). In contrast, the ROM for breast lesions regarded as suspicious or malignant using the IAC Yokohama system ranges from 84.6 to 100%. Hence, in this study, we re-classified the five categories of lesions in a two-tier class depending on the magnitude of ROM to investigate the correlation between distribution of breast lesions and their respective age groups. Breast lesions classified as malignant or suspicious were considered as ‘high-risk for malignancy (HRM)’ whereas the three categories namely atypical, benign and inadequate material were regarded as ‘low-risk for malignancy (LRM)’.

### Data collection and statistical analysis

Data gleaned from patients' medical records were available in the form of breast cytology report in the department of pathology at ENHL. The raw data were put into an Excel spreadsheet and then analyzed using Statistical Package for the Social Sciences version 23. Frequencies and percentages were calculated for categorical data. Likewise, mean, median, range and standard deviation were calculated for continuous data. Pearson Chi-squared test was used to investigate the association between distribution of breast lesions and patients’ corresponding age group. A *p* value < 0.05 was considered as statistically significant.

## Results

### Baseline characteristics of study subjects

Table 1 provides a summary of basic information about the study participants with respect to their sex and age. Of all the 905 recruited subjects, the vast majority of patients were females (96.24%) with a male to female ratio of approximately 1:26. FNAC was most frequently performed in patients within the age range of 21–30 (41.2%) and least performed in those greater than 60 years old (6.7%). The overall mean age of patients at presentation was 33.05 ± 14.9 years with age range of 80 years (range, 93–13 years). The sex-specific mean age was 32.60 ± 14.66 (range, 93–14) years for women and 45.06 ± 16.28 (range, 79–13) years for men.

**Table 1:** Sex and age distribution of study population who attended FNAC at ENHL

Variable	Frequency	Percentage (%)
Gender		
Female	871	96.24
Male	34	3.76
Total	905	100
Age group (in years)		
≤ 20	162	17.90
21–30	373	41.22
31–40	134	14.80
41–50	100	11.05
51–60	75	8.29
> 60	61	6.74
Total	905	100

### Clinical presentations of study participants

The clinical symptoms of patients are summarized in Table 2. Palpable breast mass was the most common presenting symptom (98.56%) followed by breast pain (11.82%) and presence of enlarged axillary lymph nodes on either side (3.65%). On the other hand, skin changes (1.88%) and nipple retractions (1.66%) were the least common clinical presentations. All of the male patients presented with a complaint of breast lump with or without additional symptoms. The median duration of symptoms at the time of diagnosis for all patients was seven months.

**Table 2:** Clinical presentations of study participants

Clinical symptom	Total (*n* = 905)	Percent (%)	Female (*n* = 871)	Percent (%)	Male (*n* = 34)	Percent (%)
Lump	892	98.56	859	98.62	34	100
Pain	107	11.82	99	11.37	8	23.53
Lymph node enlargement	33	3.65	32	3.67	1	2.94
Nipple discharge	29	3.20	27	3.09	2	5.88
Skin changes	17	1.88	16	1.83	1	2.94
Retraction	15	1.66	15	1.72	0	0

### Overall and sex-specific distribution of breast lesions according to specific diagnosis

The overall and sex-specific frequency distribution of breast lesions diagnosed by FNAC is depicted in Table 3. In women, fibroadenoma (39.01%) and fibrocystic breast disease (14.70%) constituted the most frequently diagnosed benign breast lesions whereas ductal carcinoma of the breast (8.73%) was the most frequently diagnosed malignancy. Other frequently diagnosed breast lesions included benign breast lesions not otherwise specified (6.19%), lipoma (4.64%), and benign proliferative breast disease (4.09%). The cytology sample was reported to be "unsatisfactory" in 11 cases and "normal finding" in one patient.

**Table 3:** Overall and sex-specific frequency distribution of breast lesions diagnosed by FNAC

DIAGNOSIS	Frequency (*n* = 905)	Percent (%)	Female *(n* = 871)	Percent (%)	Male (*n* = 34)	Percent (%)
Fibroadenoma	353	39.01	353	40.54	0	0
Fibrocystic disease	133	14.70	133	15.28	0	0
Ductal carcinoma of the breast	79	8.73	78	8.97	1	2.94
Benign breast lesion, NOS	56	6.19	52	5.97	4	11.78
Suspicious for malignancy	51	5.64	49	5.63	2	5.88
Lipoma	42	4.64	42	4.82	0	0
Benign proliferative breast disease	37	4.09	35	4.03	2	5.88
Gynecomastia	19	2.11	0	0	19	55.88
Malignant breast lesion	18	1.99	18	2.07	0	0
Fat tissue	17	1.88	15	1.72	2	5.88
Galactocele	16	1.77	16	1.84	0	0
Unsatisfactory sample	11	1.22	10	1.15	1	2.94
Benign breast lesion with atypia	10	1.10	9	1.03	1	2.94
Breast abscess	10	1.10	10	1.15	0	0
Inflammatory breast disease	9	0.99	9	1.03	0	0
Phyllodes tumor	6	0.66	6	0.69	0	0
Atypical ductal hyperplasia	5	0.55	5	0.57	0	0
Mammary duct ectasia	4	0.44	4	0.46	0	0
Non-specific finding	4	0.44	3	0.34	1	2.94
Ductal hyperplasia	3	0.33	3	0.34	0	0
Intraductal papilloma	3	0.33	3	0.34	0	0
Lobular carcinoma	3	0.33	3	0.34	0	0
Cystic breast lesion	2	0.22	2	0.23	0	0
Fat necrosis	2	0.22	2	0.23	0	0
Granulomatous mastitis	2	0.22	1	0.11	1	2.94
Inflammatory carcinoma of the breast	2	0.22	2	0.23	0	0
Acute mastitis	1	0.11	1	0.11	0	0
Colloid carcinoma of the breast	1	0.11	1	0.11	0	0
Ectopic disease of the breast	1	0.11	1	0.11	0	0
Fibroadenomatosis	1	0.11	1	0.11	0	0
Fibrolipoma	1	0.11	1	0.11	0	0
Fibroma	1	0.11	1	0.11	0	0
Normal condition	1	0.11	1	0.11	0	0
Scar tissue	1	0.11	1	0.11	0	0
Total	905	100%	871	100%	34	100%

*NOS* not otherwise specified

The two most frequent benign lesions, fibroadenoma and fibrocystic breast disease were exclusively seen in females. In the male population, gynecomastia followed by "benign breast lesion not otherwise specified" were the two leading FNAC diagnoses making up 61.29% and 12.90% of their diagnoses, respectively. Apart from ductal carcinoma, other malignant breast lesions identified were lobular carcinoma (0.33%), inflammatory carcinoma of the breast (0.22%) and colloid carcinoma (0.11%). The FNAC report in 51 (5.64%) subjects, (49 females versus 2 males) was "suspicious for malignancy." Eighteen female patients were diagnosed as having “malignant breast lesion” with no further detailed microscopic description.

### Distribution of breast lesions in symptomatic patients at ENHL according to IAC category

Distribution patterns of breast lesions in symptomatic patients were also reported against their categorical age according to IAC Yokohama classification system as shown in Table 4. Approximately 42.6% of malignant breast lesions were seen in patients older than 60 years. The age group 21–30 had the least number of malignant lesions (2.1%) while those ≤ 20 had no single malignant lesion reported. The highest proportion of benign breast lesions was observed in the age category of 20 years and below (97.5%) followed by patients within age range of 21 and 30 years (91.2%). Otherwise, there was no striking variation in the distribution of suspicious and atypical breast lesions across all age groups.

**Table 4:** Distribution pattern of breast lesions in symptomatic patients at ENHL by age group (*n* = 905)

Age group	Malignant	Suspicious	Atypical	Benign	Inadequate material	Total
≤ 20	0 (0.0%)	2 (1.2%)	1 (0.6%)	158 (97.6%)	1 (0.6%)	162 (100%)
21–30	8 (2.1%)	8 (2.1%)	12 (3.2%)	340 (91.3%)	5 (1.3%)	373 (100%)
31–40	15 (11.2%)	6 (4.5%)	6 (4.5%)	106 (79.1%)	1 (0.7%)	134 (100%)
41–50	26 (26.0%)	17 (17.0%)	10 (10.0%)	45 (45.0%)	2 (2.0%)	100 (100%)
51–60	26 (34.7%)	16 (21.3%)	7 (9.3%)	26 (34.7%)	0 (0.0%)	75 (100%)
> 60	26 (42.6%)	9 (14.8%)	3 (4.9%)	19 (31.1%)	4 (6.6%)	61 (100%)
Total	101 (11.2%)	58 (6.4%)	39 (4.3%)	694 (76.7%)	13 (1.4%)	905 (100)

*ENHL* Eritrean National Health Laboratory

### Distribution pattern of HRM and LRM breast lesions according to age group

**Fig. 1:**
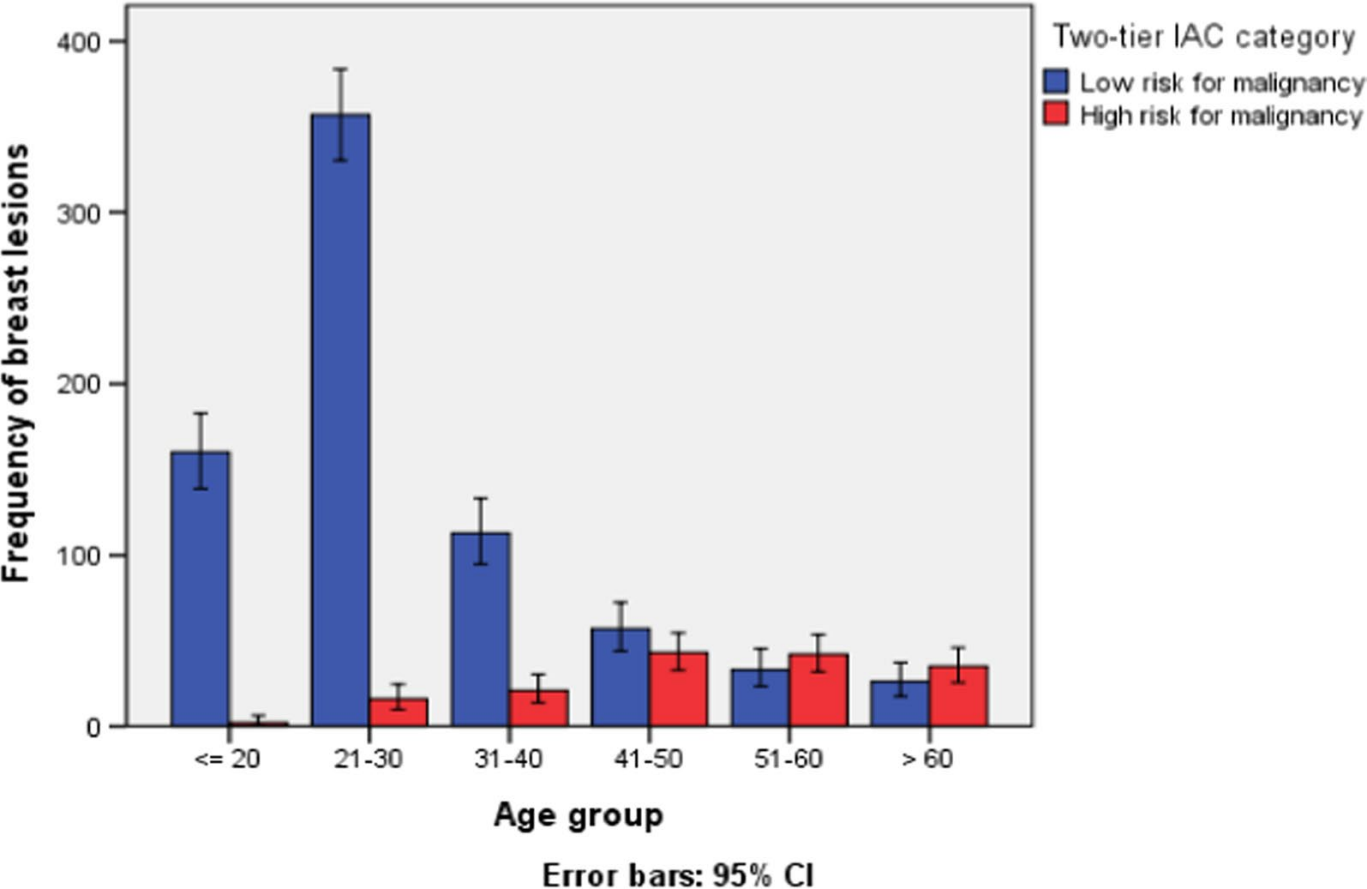
Distribution of breast lesions according to their risk for malignancy and age category

The distribution of breast lesions of varying risk for malignancy and their corresponding age groups is illustrated in Fig. 1 using the two-tier IAC category. The magnitude of high-risk for malignancy breast lesions (malignant or suspicious lesions) gradually escalated with advancing age groups reaching its highest value in the age range 41–50 years and then continued at a relatively constant pace with little decline in patients above 60 years old. Conversely, the rate of low-risk for malignancy lesions was commonly observed in the younger age groups with a maximum frequency of cases observed in the age group between 21 and 30 years (nearly 350 cases).

A Chi-squared independent test (Table 5) showed that patients with age > 40 years old were more likely to develop HRM lesions as compared to those 40 years and below (*p* < 0.001).

**Table 5:** Association between two-tier age category and risk of malignancy among study patients at ENHL

Age category	N	High risk for malignancy	Low risk for malignancy	Chi-square value	p* value
≤ 40 years	669 (73.9%)	39 (24.5%)	630 (84.5%)	244.13	< 0.001
> 40 years	236 (26.1%)	120 (75.5%)	116 (15.5%)		
Total	905 (100%)	159 (100%)	746 (100%)		

^*^Difference is significant at *p* < 0.001

### Trends in breast lesions of symptomatic women at ENHL over the study period

**Fig. 2:**
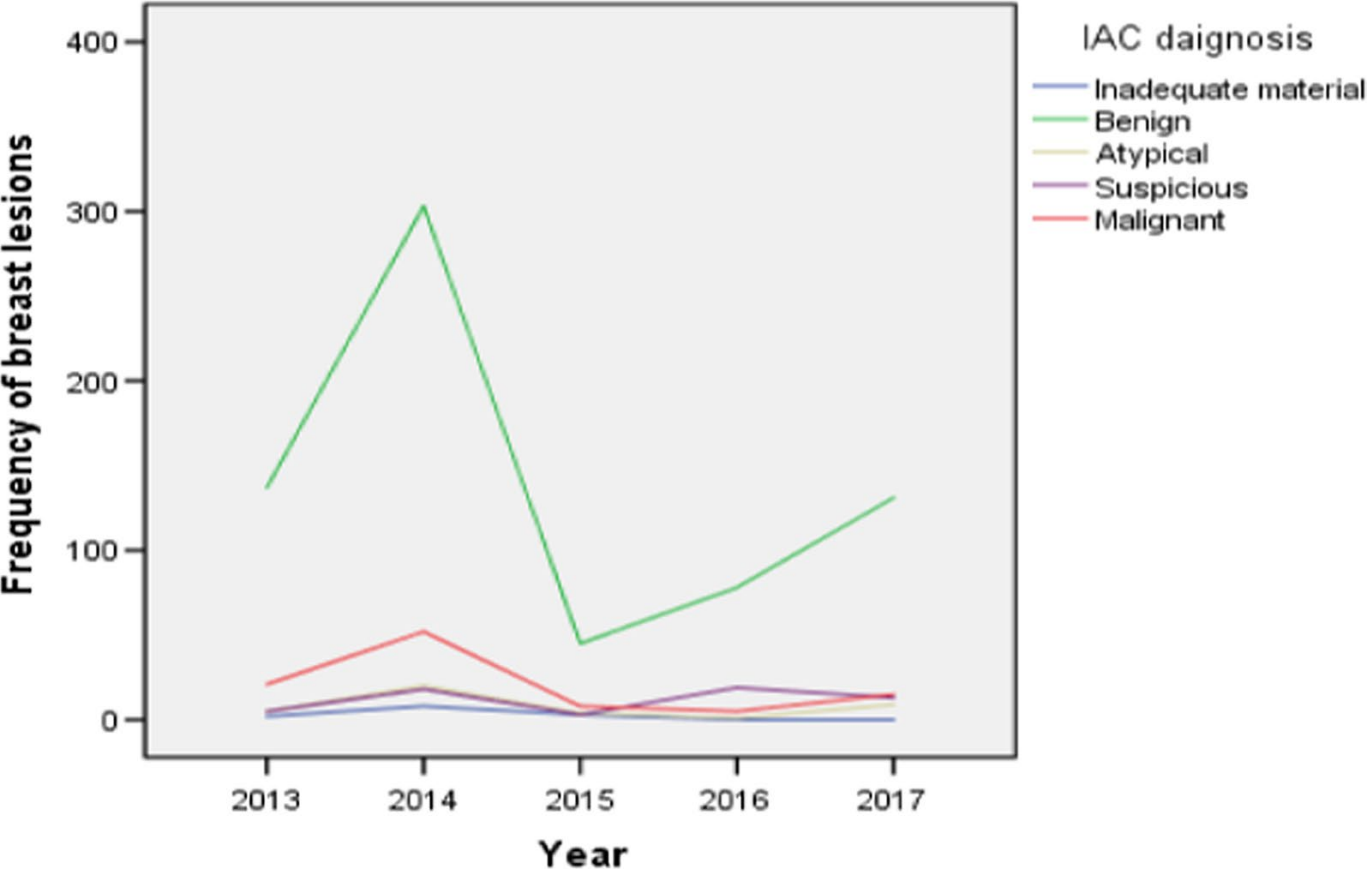
Five-year trend of breast lesions in symptomatic patients at ENHL

Overall, the highest number of FNAC in all IAC categories of breast lesions was carried out in the year 2014 and the lowest was in 2015. The fluctuations in magnitude of benign breast lesions was more striking as opposed to the rest of IAC diagnostic categories (Fig. 2). Throughout the study years, the magnitude of benign breast lesions was rising with a maximum rate of cases observed in 2014 and then sharply declined in the next year to approximately 50 cases. Later on, there was a slight elevation in the number of FNAC cases (approximately 120 cases) during the last two years of study period. The number of malignant breast lesions was second highest in magnitude during the first three consecutive years of study period after which it remained constantly low. The occurrence of suspicious and atypical lesions was relatively low, ranging from about 5 to 10 cases with little fluctuation seen across the study period.

## Discussion

The global incidence of breast cancer is increasing at an alarming rate resulting in a public health threat and adverse cosmetic impact predominantly in females [19–22]. Fine needle aspiration cytology has been established as an important tool in the evaluation of breast lesions ever since it was first introduced by Martin and Ellis in the first half of twentieth century. It has been a preferred technique of preoperative diagnosis for breast pathologies in areas where resources are limited [3, 10–12]. To the best of our knowledge, there is paucity of published data in our country that characterize the distribution of breast lesions diagnosed by FNAC. The main purpose of this study was, therefore, to describe the general distribution of breast lesions in symptomatic patients diagnosed at ENHL by FNAC over a five-year period.

In this retrospective study, out of the total 905 patients, 874 (96.6%) were females similar to studies by Otieno et al. [23] and Nwafor et al. [24], where females accounted for 98% of the study population. The median duration of disease symptoms at presentation was seven months, similar to reports from other African studies such as Ghana [25] and Kenya [23]. Delayed presentation to a health facility is not uncommon presumably as a result of social barriers, limited access to health services, lack of awareness and other individual factors which can lead to a late diagnosis thereby adversely affecting the disease outcomes [26].

Upon seeking medical help, palpable breast lump followed by mastalgia were the most frequent clinical symptoms in our patients similar to the findings of Salzman et al. [4] and Nkonge et al. [5]. Patients with breast lesions can also present with multiple symptoms simultaneously. A study in Ghana [25] showed that patients who presented with breast pain alone had a lower incidence of breast malignancy as compared to combined symptoms of pain and breast lump or nipple discharge (16% and 1.24%, correspondingly). Therefore, greater care should be taken when evaluating patients with multiple symptoms as compared to patients with a single symptom.

Proper diagnostic approach of breast lesions in cytology involves appropriate interaction between experts from multiple disciplines including medical oncology, surgery, radiology and cytopathology [9, 27]. The utilization of FNAC as an initial test accompanied by a standardized reporting system is very crucial as it maintains consistency across various practice settings. Especially, in areas where imaging facilities are not readily available, FNAC remains an ideal choice for diagnosing breast lesions and is gaining enormous popularity [5, 28, 29]. In this study, we described the distribution of breast lesions according to their specific diagnoses as well as based on the IAC Yokohama system of classification.

Considering sex-specific diagnoses, the two most frequently observed benign breast lesions in women were fibroadenoma and fibrocystic breast disease, which was in concordance with studies done by Pudasaini et al. [30], Jamal [31] and Panjvani et al. [3]. In line with these studies, our findings documented that ductal carcinoma of the breast was the most frequently diagnosed malignant breast lesion in patients above the age of 40 years. In the male population, however, gynecomastia represented the most common lesion. Hence, the similarity of our findings with those reported elsewhere suggest that the patterns of breast lesions in the Eritrean population is probably not different from the rest of the world.

After applying the IAC Yokohama system of classification, we found a significant association between increased rate of high-risk for malignancy breast lesions and age of patient greater than 40 years (*p* < 0.001). More than a quarter of all breast lesions (26%) in the age range between 41 and 50 years were found to be malignant (as shown in Table 4). Even though the exact cut-off age for ‘young women’ in breast oncology is debatable [32], a significant number of our study subjects had malignant FNAC results in their fourth and fifth decades of life. A study conducted in Spain [33] indicated that the incidence of breast cancer steadily increased among women aged 25–44 years over a period of 25 years (1980–2004). Similarly, a significant rise in frequency of malignant breast lesions in young women was reported by Bouchardy et al. between the years 1995 and 2004 (from 19.7 to 53.9 per 100,000, respectively). On the contrary, it is documented that breast malignancies are less frequent in women under the age of 40 years with overall risk reaching about 0.5% [34].

However, when breast cancer occurs in young women, it is complex and devastating with poor prognosis mainly due to risk of local and distant metastasis leading to poor patient survival [35–38]. Thus, the clinical and pathological features of breast cancers occurring in young patients are different from that diagnosed at advanced age. Breast cancers diagnosed in young women have larger tumor size, higher rate of proliferation index, increased rate of lymph node metastasis and lympho-vascular invasion as well as poorer histologic differentiation [39]. Moreover, an increased rate of a triple-negative breast cancer is observed in young women with a breast malignancy [40]. Taken together, these facts indicate that breast cancer in young women is a dire condition with not only adverse medical outcomes but also negative psychosocial repercussions as the disease may occur at the peak of their reproductive age rendering patients incapacitated with a struggle to assure balance between family life and work. In addition, they become victims of the ramifications of recurrent disease or long-term effects of therapeutic intervention. Importantly, young women with breast cancer are more likely to suffer from psychological stress owing to greater concern of their beauty [41]. To ameliorate the above-mentioned consequences of breast cancer, especially in younger women, early detection of breast lesions particularly in high risk individuals is crucial by employing regular screening programs.

The general trend of breast lesions classified by the IAC system during the study period is shown in Fig. 2. The highest magnitude for all diagnostic categories (malignant, suspicious, atypical and benign) was recorded in 2014 and the lowest number in each category was observed in 2015. We could not have any apparent reason for such fluctuations in the number of cases across the study period.

The relatively large sample size and the fact that it was conducted in a testing center that is the nation’s only site for cytological diagnosis of specimens reflect potential strengths of our study. However, limitations are yet undeniable. Owing to its retrospective nature, the extent and quality of data collected could be influenced by the nature of the record system, which was our sole source. In some cases, clinical information in the medical record were short of detail. Lack of uniformity with regard to the reporting terminologies used in the final cytological diagnosis was another limitation that could impact our findings. In order to tackle all the above-mentioned problems, it is necessary to adopt a standardized patient request form as well as standardized diagnostic categories for reporting. The use of a standardized format of cytology request form could ascertain the existence of reliable data. And more importantly, compliance with standard guidelines for reporting enhances communication within a multi-disciplinary team and assures comparison of results from other centers. The findings of our study may be used as a baseline for conducting a large-scale prospective study of the subject matter in the future.

## Conclusion

As shown in our study, FNAC can be used to filter and differentiate the various types of breast lesions in symptomatic patients. On that basis, we can conclude that FNAC is a valuable tool for evaluating palpable breast lumps in poorly-resourced healthcare settings. This study revealed that women above the age of 40 years old were more likely to develop malignant breast lesions as opposed to less than 40 years old. Delayed presentation to a health facility is commonly found, presumably attributed to lack of patients' awareness and limited access to screening facilities. To curb the negative consequences associated with delay in presentation and diagnosis of breast lesions, establishment of screening programs to detect malignant breast lesions at an early stage and fostering awareness campaigns to encourage patients seek prompt medical help is highly recommended in the setting. Finally, although FNAC is helpful to categorize the nature of breast lesions, further pathologic and other ancillary tests are necessary for a more specific diagnosis.

## Data Availability

The datasets used for this study are available from the corresponding author on reasonable request.

## Abbreviations

FNAC: Fine needle aspiration cytology
ENHL: Eritrean National Health Laboratory
NOS: Not otherwise specified
IAC: International Academy of Cytology
ROM: Risk of malignancy
HRM: High risk for malignancy
LRM: Low risk for malignancy
